# Algorithmic Opacity, Clinical Justification, and the Duty of Candour in AI-Assisted Care

**DOI:** 10.7759/cureus.113364

**Published:** 2026-07-25

**Authors:** Hanan Ibrahim, Zack Hudson

**Affiliations:** 1 Stroke, Sandwell & West Birmingham Hospitals NHS Trust, Birmingham, GBR

**Keywords:** accountability, algorithims, artificial intelligence in medicine, decision-support tool, duty of candour, duty of care, ethics in ai, patient-centred care, safety and health, triage

## Abstract

Artificial intelligence (AI) systems are becoming increasingly embedded within NHS clinical systems, including areas such as emergency department triage, diagnostic support, and risk stratification. Many high-performing systems rely on complex machine learning architectures that are difficult or even impossible for clinicians to interpret at the point of care. This paper aims to examine the ethical implications of deploying uninterpretable AI systems in high-risk clinical environments. Utilising a constructed, however clinically plausible, vignette of sepsis misclassification, we argue that algorithmic opacity may undermine clinicians' ability to satisfy justificatory obligations within the professional and legal framework of the UK Duty of Candour. We distinguish between ordinary clinical uncertainty and structural opacity arising from system design, arguing that the latter generates a distinctive ethical problem by weakening traceability, accountability, and meaningful explanation after patient harm. We further argue that interpretability should be treated as a safety-relevant ethical requirement in high-risk clinical contexts, rather than as an optional technical feature subordinate to predictive performance alone.

## Introduction

Artificial intelligence (AI)-enabled clinical decision support systems are increasingly integrated into healthcare delivery across the NHS. Applications now include radiological interpretation, deterioration prediction, sepsis alerts, and emergency department triage. These systems are frequently justified on the basis of improved efficiency, scalability, and predictive performance. However, many contemporary machine learning systems, particularly deep neural networks, operate through computational processes that are opaque to human users [1].

This creates what may be termed a 'Transparency Paradox': the unsettling reality that as our clinical tools become more statistically powerful, they become less humanly comprehensible. As predictive systems become more sophisticated and statistically performant, the capacity of clinicians to interrogate and explain individual outputs may diminish. While this problem is often discussed in technical terms under the heading of “explainable AI” (XAI), its implications extend beyond engineering. In medicine, clinicians are not merely expected to produce outcomes; they are also expected to justify decisions, explain reasoning, and remain accountable for patient care.

The ethical significance of this issue becomes particularly acute within the UK context, where clinicians operate under legal and professional obligations requiring openness following patient harm. Under the statutory Duty of Candour, healthcare organisations must provide patients with truthful accounts of safety incidents and meaningful explanations where possible [2]. Simultaneously, professional guidance from the General Medical Council (GMC) maintains that clinicians remain responsible for decisions made with the assistance of digital systems [3].

This paper argues that deploying non-interpretable AI systems in high-risk clinical contexts creates a presumption of ethical impermissibility where such systems foreseeably undermine clinicians’ ability to fulfil justificatory obligations to patients. The central problem is not simply that AI systems can make mistakes. Rather, it is that some systems are designed in ways that prevent meaningful retrospective explanation when mistakes occur.

While existing debates on explainable AI in healthcare have largely focused on technical transparency, patient trust, or model optimisation, less attention has been given to how algorithmic opacity intersects with these existing medico-legal frameworks. This paper reframes interpretability not merely as a technical design preference but as a context-dependent ethical requirement linked to the normative structure of clinical responsibility.

Methodological approach

To address these issues, this paper employs a normative-analytical methodology that integrates legal analysis of UK jurisprudence with clinical ethics. Our analysis is structured around three core components:

Literature and Legal Source Selection

We conducted a targeted, iterative literature search across PubMed, Google Scholar, and Westlaw UK. Legal analysis focused on seminal UK Supreme Court and appellate-level healthcare jurisprudence (most notably Montgomery v Lanarkshire [4]). Medical, ethical, and regulatory literature was selected based on its direct relevance to clinical decision-support systems, professional accountability, and the UK Duty of Candour.

Vignette Construction

To ground our theoretical analysis in frontline realities, we construct a clinical vignette of a high-stakes acute sepsis scenario. The clinical parameters and failure modes depicted in this vignette, including algorithmic alert thresholds and opaque proprietary feature weights, are not speculative; they are explicitly mapped to documented technical limitations, high false-alarm rates, and validation challenges of widely deployed real-world sepsis prediction models.

Analytical Framework

Our ethical evaluation is guided by a risk-stratified analysis. We evaluate the Transparency Paradox by mapping levels of technical opacity against the clinical risk tier of the decision-support tool, assessing how this relationship impacts a clinician's capacity to fulfil their professional obligations.

Specifically, this paper addresses three central objectives: first, we examine how structural algorithmic opacity disrupts the clinician's capacity to fulfil their professional Duty of Candour; second, we analyse the extent to which the clinical risk profile of an AI tool dictates its required level of interpretability; and finally, we explore how UK medico-legal principles, such as those established in Montgomery v Lanarkshire, can inform a risk-stratified framework for clinical AI deployment. 

## Materials and methods

The anatomy of algorithmic opacity: a clinical case study

To illustrate this tension, consider a constructed but clinically plausible scenario informed by known limitations of machine learning-based deterioration prediction systems.

A 58-year-old woman presents to an emergency department with malaise, lethargy, and mild confusion. Her physiological observations are borderline abnormal but do not trigger conventional escalation thresholds. An AI-supported triage system trained on retrospective electronic health record data classifies the patient as low risk for sepsis-related deterioration. Consequently, escalation to senior clinical review is deferred in favour of patients assigned higher acuity scores.

Several hours later, the patient deteriorates rapidly and develops septic shock. Subsequent review identifies long-term beta-blocker therapy, which attenuated tachycardia, a variable heavily weighted within the model’s learned representation of sepsis risk.

During post-incident investigation, clinicians are unable to determine why the system discounted neurological symptoms such as confusion or how specific variables contributed to the low-risk classification. The software vendor cites proprietary constraints and declines to disclose the underlying model architecture or weighting mechanisms.

This vignette is not intended to demonstrate that AI systems are uniquely error-prone. Rather, it highlights a distinctive ethical problem: when opaque systems materially influence clinical decisions, the capacity to provide meaningful justification after patient harm may become structurally compromised.

The Duty of Candour and the ethics of explanation

Under Regulation 20 of the Health and Social Care Act 2008 [2], healthcare providers must act in an open and transparent manner following notifiable patient safety incidents. This includes informing patients about what occurred, offering an apology, and providing an account of known facts.

Importantly, the Duty of Candour does not require absolute certainty or omniscience. Clinical medicine routinely involves uncertainty, probabilistic reasoning, and incomplete information. However, ethical and professional obligations in medicine extend beyond disclosure of outcomes alone. They also involve what may be termed justificatory accountability: the ability to explain how decisions were reached and why particular actions were taken.

A critical distinction, therefore, emerges between epistemic limitation and structural opacity. Epistemic limitation refers to scenarios where clinicians do not yet fully understand why an outcome occurred, despite the decision-making process remaining in principle interrogable. Conversely, structural opacity describes systems designed in ways that fundamentally preclude meaningful explanation or traceability.

Crucially, we must define what constitutes a 'meaningful explanation' in the context of clinical AI. Within a high-stakes clinical workflow, a meaningful explanation does not require a complete mathematical decryption of a deep neural network's node weights, nor does it demand that a clinician explain complex statistical code. Rather, it must translate algorithmic behaviour into a clinically intelligible account. To satisfy this standard, the explanation must provide three key components: (1) the primary clinical features (such as specific vital signs, laboratory trends, or demographic inputs) that drove the system's output; (2) the underlying clinical reasoning or decision-logic of the inference represented in medical terms; and (3) the model's known boundaries of reliability or error rates for patients of that specific clinical presentation.

This distinction is ethically significant because uncertainty is an enduring feature of medicine, whereas structural opacity is an engineered design property. In the latter case, the inability to provide an explanation is not accidental or temporary, but intrinsic to the architecture of the system itself. In the sepsis vignette described above, clinicians could disclose the adverse outcome itself, yet remained unable to provide a meaningful account of how the triage recommendation had been generated or weighted. The ethical concern therefore extends beyond uncertainty regarding prediction and toward impairment of retrospective justification itself.

Several scholars have argued that explanation in healthcare serves morally important functions beyond technical transparency alone. Wachter, Mittelstadt, and Russell note that explanation supports accountability and contestability even where full algorithmic transparency is unattainable [5]. Similarly, Babic and colleagues caution against superficial conceptions of explainability that fail to address how clinicians and patients meaningfully understand AI-assisted decisions [6].

Within clinical care, explanation is closely connected to respect for patient autonomy. Patients are not merely passive recipients of outcomes; they are moral agents entitled to intelligible reasons for decisions affecting their welfare [4]. Where clinicians cannot explain why a harmful recommendation emerged from a system materially influencing care, the justificatory relationship between clinician and patient may be weakened.

The importance of intelligible explanation within clinical care is also reflected in contemporary medico-legal developments surrounding informed consent. In Montgomery v Lanarkshire Health Board, the UK Supreme Court emphasised the importance of dialogue and material understanding in patient decision-making, rejecting excessively paternalistic models of disclosure [7]. Although Montgomery concerned informed consent rather than AI-assisted decision-making specifically, its emphasis upon intelligible explanation reinforces the broader ethical significance of justificatory communication within clinical practice.

Professional responsibility and automation bias

Professional guidance consistently maintains that clinicians remain responsible for decisions made with the assistance of AI systems [3]. Yet this principle becomes ethically strained where clinicians cannot realistically interrogate system outputs.

Research in cognitive psychology and human factors demonstrates the phenomenon of automation bias, which is characterised by the tendency for individuals to over-rely on automated recommendations, particularly under conditions of cognitive load and time pressure [8]. Recent empirical work has demonstrated the persistence of this phenomenon across modern clinical specialities. For instance, a study in computational pathology found that even highly trained experts suffered a measurable automation bias rate under acute time constraints, actively overturning their own correct pathological assessments in favour of erroneous AI diagnostic cues [9]. Similarly, contemporary clinical trials of AI-enabled decision support systems show that a user's perceived benefit of a system strongly correlates with false agreement rates, with non-specialists remaining the most vulnerable to blindly accepting erroneous algorithmic classifications.

Opaque systems may exacerbate automation bias through multiple mechanisms. They reduce opportunities for critical interrogation because clinicians cannot readily examine the basis of recommendations, and opacity may simultaneously create exaggerated perceptions of objectivity and reliability associated with computational systems. Furthermore, a lack of interpretability weakens opportunities for reflective learning following error.

The constructed vignette also illustrates how opacity may interact with automation bias under conditions of emergency department time pressure. Because clinicians lacked access to intelligible reasoning pathways, opportunities for critical challenge of the low-risk classification were substantially reduced.

Importantly, the ethical concern is not that clinicians use decision support systems per se. Medicine has long incorporated probabilistic tools and clinical scoring systems. Rather, the concern arises when systems influence care while simultaneously resisting meaningful scrutiny. This creates what has been described in AI ethics literature as a responsibility gap, representing a situation in which harmful outcomes occur, yet no actor can adequately explain or justify how the relevant decision process unfolded [10]. In healthcare contexts grounded in professional accountability, such gaps are ethically problematic.

Where opaque systems materially influence care pathways, uncertainty may also arise regarding the distribution of liability between clinicians, healthcare institutions, and commercial developers, particularly when proprietary protections limit external scrutiny of system behaviour.

Counterargument: is explainability always necessary?

One possible objection is that interpretability should not be privileged over clinical performance. If opaque systems demonstrably improve patient outcomes at a population level, restricting their use on explainability grounds may itself be ethically harmful.

This objection deserves serious consideration. Human clinical reasoning is often imperfect, inconsistent, and partially opaque even to clinicians themselves. Moreover, highly interpretable models may underperform relative to more complex architectures in some predictive tasks [11]. It could therefore be argued that insisting upon interpretability risks sacrificing accuracy for transparency. However, this objection does not fully resolve the ethical problem in high-risk clinical settings.

First, the issue is not whether clinicians can achieve perfect explanation, but whether systems permit meaningful retrospective justification. Human decision-making, while imperfect, remains open to interrogation through testimony, reasoning, contextual analysis, and professional reflection. By contrast, structurally opaque systems may render certain forms of explanation fundamentally inaccessible.

Second, ethical permissibility in medicine cannot be reduced solely to outcome optimisation. Clinical practice is grounded not only in beneficence but also in accountability, autonomy, and trust [12]. A system that improves aggregate predictive performance while undermining the conditions necessary for meaningful explanation may generate ethically relevant harms even when statistically advantageous.

Third, the ethical weight of explainability is context-dependent. In low-risk administrative tasks, for instance, an AI tool designed to optimise clinic scheduling by predicting patient did-not-attend (DNA) rates may operate on a highly complex, opaque ensemble model. If the system misclassifies a patient's likelihood of missing an appointment, the worst-case outcome is a minor scheduling inefficiency or a redundant automated text reminder. Because this administrative error carries no risk of physiological deterioration, diagnostic delay, or physical harm, the clinician’s justificatory burden is virtually non-existent, rendering the model's structural opacity ethically trivial compared to an acute triage algorithm. Opacity may be acceptable. In high-stakes triage or diagnostic contexts, however, the justificatory burden is substantially higher because decisions directly affect patient safety and resource prioritisation.

Practical deployment decisions inevitably involve trade-offs between interpretability, predictive performance, procurement constraints, and commercial considerations. In some cases, highly performant models may offer clinically meaningful benefits despite limited transparency. The argument advanced here is therefore not that opacity automatically renders systems impermissible, but that increasing clinical risk generates correspondingly stronger justificatory obligations regarding auditability, explanation, and contestability. Ethical evaluation should consequently consider not only aggregate predictive performance, but also whether systems preserve the practical conditions necessary for professional accountability following patient harm. Where explanation is structurally unavailable, accountability may become structurally weakened.

## Results

The regulatory landscape and the interpretability deficit

UK regulatory approaches to healthcare AI continue to evolve through guidance from the Medicines and Healthcare products Regulatory Agency, the National Institute for Health and Care Excellence, and the Department of Health and Social Care. Current frameworks increasingly emphasise safety assurance, post-market surveillance, and dataset validation [13-14].

However, a gap persists between performance validation and bedside interpretability. Existing evaluation pathways focus primarily on sensitivity and specificity, dataset quality and representativeness, technical robustness, and generalisability. Conversely, substantially less attention is given to whether systems support meaningful clinician interrogation, case-level explanation, post hoc investigation following harm, or the practical fulfilment of professional justificatory duties.

This creates a potential misalignment between regulatory approval standards and the ethical realities of clinical accountability. Notably, the sepsis vignette described above could satisfy existing requirements for technical validation while still leaving clinicians unable to reconstruct or meaningfully explain the basis of a harmful recommendation at a case level. Interpretability should therefore be understood not merely as a technical preference, but as a safety-relevant ethical property whose importance varies according to clinical risk.

## Discussion

A path forward: a risk-stratified framework for clinical AI

The findings of this normative analysis indicate that evaluating clinical AI solely through population-level predictive metrics creates a critical deficit in bedside accountability. To bridge this interpretability gap, we propose moving away from viewing explainability as either universally mandatory or entirely dispensable, pivoting instead toward a risk-stratified framework where interpretability is mandated as a safety-relevant ethical property. This aligns with recent empirical evaluations of clinical alert systems, such as the Epic sepsis model, where real-world deployment exposed substantial hidden failure modes due to localised data confounding and a lack of user-facing transparency [15]. When an algorithmic output cannot be critically cross-examined at the bedside, clinicians face an ethically challenging choice between blind compliance and complete rejection, exacerbating the risks of automation bias [8].

Under our proposed framework, high-risk systems operating in critical care, acute diagnostics, or emergency triage must incorporate robust explanatory functionality, clinically intelligible reasoning pathways, and case-level auditability. This position is supported by recent work by Amann et al., who argue that explainability is an essential component of clinical trust and ethical validation in digital health [16]. Similarly, the legal analysis by Price establishes that black-box algorithms complicate traditional medical malpractice frameworks because they obscure whether a diagnostic error originated from user negligence or a software defect [17]. By insisting on actionable, human-accessible reasoning pathways for high-acuity tools, healthcare organisations can ensure that the legal protections afforded by the statutory Duty of Candour remain intact [2].

Conversely, the stringency of these interpretability demands should vary dynamically based on contextual risk parameters. These parameters include the severity of potential clinical harm, the degree of clinical autonomy retained by the user, the reversibility of the resulting decisions, and the inherent vulnerability of the affected patient population. For instance, low-risk administrative scheduling software or automated billing codes require minimal interpretability, as standard technical validation suffices. Moderate-risk tools, such as chronic disease monitoring platforms or outpatient scheduling prioritisation systems, require partial explainability, which can be satisfied via local feature-importance metrics like SHAP (SHapley Additive exPlanations) or LIME (Local Interpretable Model-agnostic Explanations) values.

However, a critical caveat must be acknowledged: post-hoc explanation methods like SHAP and LIME are not a technical silver bullet. Because these tools construct simplified approximations of highly complex, non-linear decision boundaries, they can be highly unstable, unfaithful to the underlying model, or even mathematically manipulated. Relying on them uncritically introduces distinct epistemic risks-specifically a fidelity-interpretability trade-off-which can inadvertently foster a false sense of security among clinicians who mistake a localised approximation for the actual computational reality of the model [18].

High-risk diagnostic or emergency triage systems must be subjected to the highest standards of robust explanatory functionality as an explicit condition of clinical deployment.

Finally, resolving the responsibility gaps introduced by clinical AI requires a model of shared accountability distributed across frontline clinicians, healthcare institutions, and commercial developers. Forcing clinicians to carry the entire liability burden for uninterpretable systems is unsustainable and legally incoherent under current UK professional standards [3]. This distributed approach mirrors the governance models advocated by Sullivan and Schweikart, who stress that product liability frameworks must adapt to share the defensive burden with commercial software vendors when proprietary designs impede post-incident investigations [19]. When a commercial system materially shapes a care pathway, the vendor must bear a corresponding legal obligation to provide the software utilities necessary to support comprehensive retrospective audits following an adverse event.

Limitations of the study

While this paper offers a rigorous normative-analytical framework for navigating the intersection of clinical AI and the statutory Duty of Candour, several key limitations of our analysis must be acknowledged.

First, our analysis is fundamentally interpretive and policy-oriented rather than empirical. We do not present direct observational data from live clinical trials, nor does this paper incorporate qualitative stakeholder evidence-such as direct surveys or structured interviews exploring the perspectives of frontline NHS clinicians, patients, healthcare managers, or defence union representatives. Consequently, our findings should be understood as a normative roadmap for governance and ethical design rather than an empirical assessment of current clinical behaviours.

Second, although our literature review and selection of legal precedents were highly targeted and iterative-specifically focusing on authoritative UK Supreme Court and appellate-level jurisprudence (such as Montgomery v Lanarkshire)-this research does not constitute a formal systematic review. There remains a possibility that peripheral regulatory guidelines, regional NHS trust policies, or emerging lower-court rulings relevant to AI liability were not fully captured in our analysis.

Third, our legal and regulatory analysis is intentionally situated within the specific context of UK healthcare law and professional guidance (specifically from the GMC and the Care Quality Commission). While the ethical principles underpinning our risk-stratified framework are broadly applicable, the specific legal obligations discussed-including the statutory Duty of Candour-limit the direct generalizability of our conclusions to jurisdictions with vastly different liability, regulatory, and malpractice frameworks (such as the United States or member states of the European Union).

Finally, our proposed risk-stratified framework is conceptual. While we define the qualitative parameters of "meaningful explanation" and map them to clinical risk tiers, this paper does not attempt to define the precise mathematical or technical thresholds of interpretability (such as specific local feature attribution values or SHAP/LIME metrics) required for clinical software certification.

Directions for future research

To address these limitations, future interdisciplinary research must bridge the gap between normative theory and clinical practice. Priority should be given to conducting qualitative empirical studies with NHS clinicians to assess how they perceive their own liability and professional duties when acting on opaque algorithmic recommendations. Additionally, patient-centred cohort studies are essential to investigate public expectations regarding what information should be disclosed about AI involvement in care, particularly following an adverse event or misdiagnosis. Finally, close collaboration between bioethicists, clinicians, and computer scientists is required to translate our qualitative, risk-stratified demands into concrete, standardised technical specifications for medical AI developers during the software design phase.

Practical implications for clinicians and policymakers

While our findings are normative rather than empirical, they offer immediate practical guidance for key healthcare stakeholders.

For clinicians, this framework reassures frontline practitioners that they are not expected to understand the complex mathematical inner workings of deep-learning tools. Instead, it empowers them to demand "meaningful explanations"-clinically intelligible summaries of key feature weights and reliability boundaries-before deploying high-risk tools on the ward, thereby safeguarding their ability to fulfil their professional Duty of Candour.

For healthcare policymakers and trusts, rather than implementing blanket transparency rules that risk stalling NHS innovation, policymakers should adopt a risk-stratified procurement model. Low-risk applications (e.g., administrative triage) can safely utilise high-performing, opaque "black-box" systems. Conversely, high-risk systems (e.g., acute sepsis prediction) must have explainability and auditability built into their design and procurement specifications as a non-negotiable ethical and legal requirement.

## Conclusions

AI systems offer substantial opportunities to improve healthcare delivery within the NHS. However, the ethical acceptability of these systems cannot be assessed solely through aggregate measures of predictive performance. In medicine, clinicians are required not only to act, but also to justify, explain, and remain accountable for decisions affecting patients. The Transparency Paradox identifies a growing tension within AI-assisted care: systems optimised for predictive performance may simultaneously weaken the justificatory conditions underpinning professional accountability and patient trust. This paper has argued that structural opacity differs ethically from ordinary clinical uncertainty because it represents a design feature that can foreseeably impede meaningful explanation after patient harm.

This paper does not argue that all healthcare AI systems must be fully transparent or technically interpretable in every context. Rather, the goal is not to stifle AI innovation within the NHS, but to ensure its safe and sustainable integration. We must treat clinical interpretability not as an optional, high-tech add-on, but as a fundamental, non-negotiable safety feature-much like a car's brake indicators. Just as we would never deploy high-speed vehicles on public roads without functional brake lights to signal their operations to others, we cannot safely integrate highly predictive clinical models into acute medicine without an equivalent mechanism of transparency. Therefore, in high-risk clinical environments, there are ethical limits to acceptable opacity. While a low-risk administrative tool (such as a scheduling prediction algorithm) can operate opaquely without causing moral harm, high-stakes systems that directly steer acute clinical outcomes demand a different standard. JJust as established legal precedents mandate patient-centered, intelligible communication regarding clinical risks, a parallel ethical duty exists to ensure that high-stakes algorithmic decisions remain clinically explainable. Where systems materially influence triage, diagnostic prioritisation, or escalation decisions while simultaneously preventing meaningful retrospective explanation, the justificatory foundations of professional accountability may be weakened. Under such conditions, opacity ceases to be merely a technical characteristic and becomes an ethically significant design constraint relevant to the permissibility of clinical deployment itself.
